# Global Reprogramming of Transcription in Chinese Fir (*Cunninghamia lanceolata*) during Progressive Drought Stress and after Rewatering

**DOI:** 10.3390/ijms160715194

**Published:** 2015-07-06

**Authors:** Ruiyang Hu, Bo Wu, Huiquan Zheng, Dehuo Hu, Xinjie Wang, Hongjing Duan, Yuhan Sun, Jinxing Wang, Yue Zhang, Yun Li

**Affiliations:** 1 National Engineering Laboratory for Tree Breeding, College of Biological Sciences and Technology, Beijing Forestry University, Beijing 100083, China; E-Mails: hury1102@163.com (R.H.); wu654541823@126.com (B.W.); duan673356712@126.com (H.D.); syh831008@163.com (Y.S.); xing521_zi@163.com (J.W.); yueslife@live.cn (Y.Z.); 2 Guangdong Academy of Forestry, Guangzhou 510520, China; E-Mails: zhenghq@sinogaf.cn (H.Z.); hudehuo@163.com (D.H.); 3 College of Forestry, Beijing Forestry University, Beijing 100083, China; E-Mail: xinjiew@bjfu.edu.cn

**Keywords:** *Cunninghamia lanceolata*, drought stress, transcriptome, gene expression, rewatering

## Abstract

Chinese fir (*Cunninghamia lanceolata*), an evergreen conifer, is the most commonly grown afforestation species in southeast China due to its rapid growth and good wood qualities. To gain a better understanding of the drought-signalling pathway and the molecular metabolic reactions involved in the drought response, we performed a genome-wide transcription analysis using RNA sequence data. In this study, Chinese fir plantlets were subjected to progressively prolonged drought stress, up to 15 d, followed by rewatering under controlled environmental conditions. Based on observed morphological changes, plantlets experienced mild, moderate, or severe water stress before rehydration. Transcriptome analysis of plantlets, representing control and mild, moderate, and severe drought-stress treatments, and the rewatered plantlets, identified several thousand genes whose expression was altered in response to drought stress. Many genes whose expression was tightly coupled to the levels of drought stress were identified, suggesting involvement in Chinese fir drought adaptation responses. These genes were associated with transcription factors, signal transport, stress kinases, phytohormone signalling, and defence/stress response. The present study provides the most comprehensive transcriptome resource and the first dynamic transcriptome profiles of Chinese fir under drought stress. The drought-responsive genes identified in this study could provide further information for understanding the mechanisms of drought tolerance in Chinese fir.

## 1. Introduction

The natural environment for plants is composed of a complex set of abiotic and biotic stresses. Drought stress is an abiotic stress and it is one of the main factors that affects tree growth, vitality, and survival [1,2,3,4,5]. This is particularly the case in arid and semiarid regions. To improve the tolerance of trees to drought stress, it is essential to understand how plants respond to water deficiency and which genes and biological pathways are involved in the response [6]. Thus, an increasing number of studies have focused on the mechanisms enabling plant adaptation to dehydration [7,8,9,10].

Drought stress of plants induces a range of physiological and biochemical responses at the cellular and whole-organism levels. These responses include stomatal closure, repression of cell growth and photosynthesis, and activation of respiration [11,12]. Plants also respond and adapt to water deficit at both the cellular and molecular levels, for example, by the accumulation of osmolytes and proteins specifically involved in stress tolerance [13].

One of the most active fields of plant science research focuses on the understanding of genetic responses to drought stress, and the development of approaches to improve tolerance and acclimation [14]. Many genes respond to drought at the transcriptional level [15,16,17]. Several drought-inducible genes and their products, such as the phosphatidylinositol synthase gene (*ZmPIS*) [18] and the transcription factor (TF) DREB1A [19], have been used to improve the stress tolerance of plants by gene transfer.

Although hundreds of genes have been found to be involved in drought-stress responses, and a number of them have been well characterized, the function of the majority of the genes remains unknown. There are likely many more genes to be discovered [20,21,22]. Recent advances in biotechnology have dramatically enhanced gene discovery and functional genomics [23,24,25,26]. The transcriptome represents a comprehensive set of transcribed regions throughout the genome [27]. Studying transcriptome dynamics provides important insights into functional elements of the genome, their expression patterns, and regulation of transcribed regions under drought-stress conditions [27]. Transcriptome analysis has been used extensively to identify drought-responsive genes in many plant species, and notably the application of transcriptome sequencing (RNA-Seq) has greatly improved the throughput of gene expression profiling in drought-stressed individuals. Comparison of plant profiles representing well-watered and drought-stressed treatments facilitates identification of differentially expressed genes (DEGs) [28,29]. To gain a comprehensive perspective of the molecular mechanisms and related genes underlying drought tolerance, one of the most effective methods is evaluation of genome-wide transcriptional patterns under drought stress [15]. Expression analysis results can be used to identify candidate genes for specific drought tolerance-related traits or physiological mechanisms associated with stress-induced adaptive processes [30].

The molecular aspects of drought response have been extensively investigated in *Arabidopsis thaliana* L., maize (*Zea mays* L.), rice (*Oryza sativa* L.), wheat (*Triticum aestivum* L.) and tomato (*Solanum lycopersicum* L.), yet our understanding of the molecular mechanisms underlying drought tolerance in conifers is limited due to long generation times, large genomes, and the lack of well characterized mutations for forward genetic analyses [10,31,32,33,34,35]. When confronted with water limitations, plants actively reprogram their metabolism and growth. It has become clear that plants show specific and highly dynamic responses to drought. These responses are affected by genotypes, experimental methods, and sampling times [36,37,38].

Chinese fir (*Cunninghamia lanceolata* [Lamb.] Hook) is an evergreen conifer. It is the most commonly grown afforestation species in southeast China, due to its rapid growth and good wood qualities [39]. However, the extremely harsh weather conditions typical of southern China, coupled with environmental changes (e.g., continuous drought) resulting from global warming, have caused widespread death of Chinese fir plantlets. Annual plantlets only barely survive long-term drought stress at the beginning of afforestation. It is important to develop our knowledge of the mechanism underlying the response of Chinese fir to drought stress. Previous studies focused mainly on the physiological and biochemical basis of drought-stress tolerance of Chinese fir plantlets. To date, knowledge of the transcriptomes of drought-stressed Chinese fir remains poor [40,41]. The identification of drought response genes for Chinese fir would provide genes that can be scored for variants in a population that correlates with drought tolerance, facilitating marker-assisted selection.

To identify genes that may be involved in the molecular mechanisms underlying the response of Chinese fir to drought, we used transcriptome sequencing as a tool to identify drought-responsive genes in this species. We subjected 12-month-old Chinese fir plantlets to progressively prolonged drought treatments and rewatering. Changes in gene transcript levels were measured at multiple time points during drought stress, over a 15-days period. We will present a comprehensive overview of the transcriptome changes associated with the progression of drought stress. Our goal was to identify candidate genes that might be useful in translational approaches to improvement of Chinese fir.

## 2. Results

Our experimental setup was designed to impose a progressive drought stress to mimic the situation experienced by plants in the field. Gradual application of water deficit enables the plant to adjust its metabolism and deploy its adaptive responses [42]. Based on three representative phenotypic characteristics that result from withdrawal of water, we defined the following three levels of drought stress (Figure S1): Mild stress (5-days water deficit) in which the colour of a few stem tip needles becomes light green and they dry up; moderate stress (10-days water deficit) in which the surface of the top needle shrinks and loses elasticity; and severe stress (15-days water deficit) in which most of the needles become curly and folded. At 24 h post-rewatering, the plantlets were able to fully recover from severe stress upon rehydration. Many DEGs were detected in this study using this time-course design. Their expression was affected by the duration and severity of drought, and by re-watering. This further supports the existence of a high-level drought tolerance in Chinese fir.

### 2.1. High-Throughput Transcriptome Sequencing and Read Assembly

With the purpose of screening the specifically expressed unigenes involved in Chinese fir drought stress, a cDNA sample was prepared from an equal mixture of total RNA isolated from leaves for five libraries, corresponding to the well-watered plantlets [CK], five days of drought [C], ten days of drought [D], fifteen days of drought [E], and one day post-rewatering [F] after fifteen days of drought, which were sequenced using the Illumina HiSeq™ 2000 machine (Illumina Inc., San Diego, CA, USA). About 239 million raw reads were obtained from the three drought treatments (C, D, E), 56 million from the CK treatment, and 74 million from the re-watering treatment (F). We discarded low-quality reads, which contained adapters and unknown or low-quality bases, and then removed ribosomal RNA using the Chinese fir total rRNA as a template. After data cleaning, we obtained 371,387,986 raw reads. The average read size was 90 bp. Q20 percentage (sequencing error rate < 1%) was 97.2%. GC (guanine + cytosine) content was 39.4%. With paired-end joining and gap-filling, 120,924 contigs, average length of 613 bp, were assembled into 77,229 scaffolds with an average length of 850 bp. After local assembly with the unmapped ends to fill in the small gaps within the scaffolds, the *De novo* assembly yielded 75,412 unigenes with an average length of 867 bp (Tables S1 and S2).

### 2.2. Gene Annotation and Functional Classification

To validate and annotate the assembled unigenes, we searched similar sequences against the Swiss-Prot protein database using the BLASTx algorithm with an *E*-value threshold of 10^−5^. The results indicated that, among the 75,421 unigenes, 27,634 (36.64%) had a significant similarity to known proteins (Table S3). Due to the lack of sufficient Chinese fir genomic and EST information, 63.36% of the unigenes cannot be matched to known genes.

**Figure 1:**
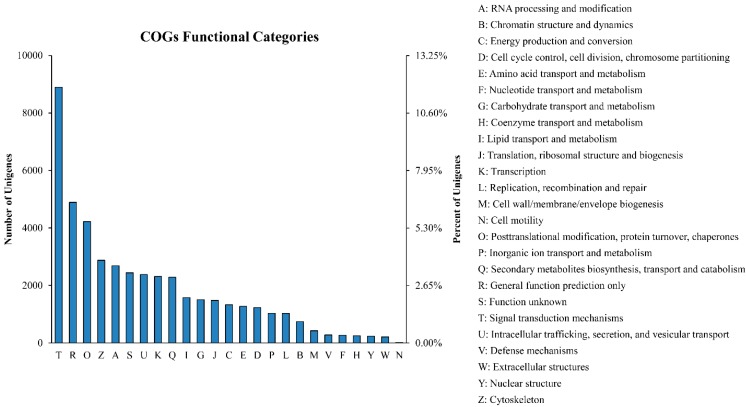
Clusters of orthologous groups (COG) classifications in Chinese fir. These 15,662 sequences have a COG classification within the 25 categories.

Among the 27,634 Swiss-Prot hits, 15,662 sequences had a COG classification. Among the 25 COG categories, the cluster for “Signal transduction mechanisms” (8896, 11.80%) represented the largest group, followed by “General function prediction only” (4890, 6.48%) and “Posttranslational modification, protein turnover, chaperones” (4225, 5.60%). The “Extracellular structures” (210, 0.28%) and “Cell motility” (7, 0.01%) categories represented the smallest groups (Figure 1). Compared with the CK sample, the largest cluster in the drought-treated samples was “Signal transduction mechanisms”, which means that these genes may play a key regulatory role in the Chinese fir drought-stress response.

**Figure 2:**
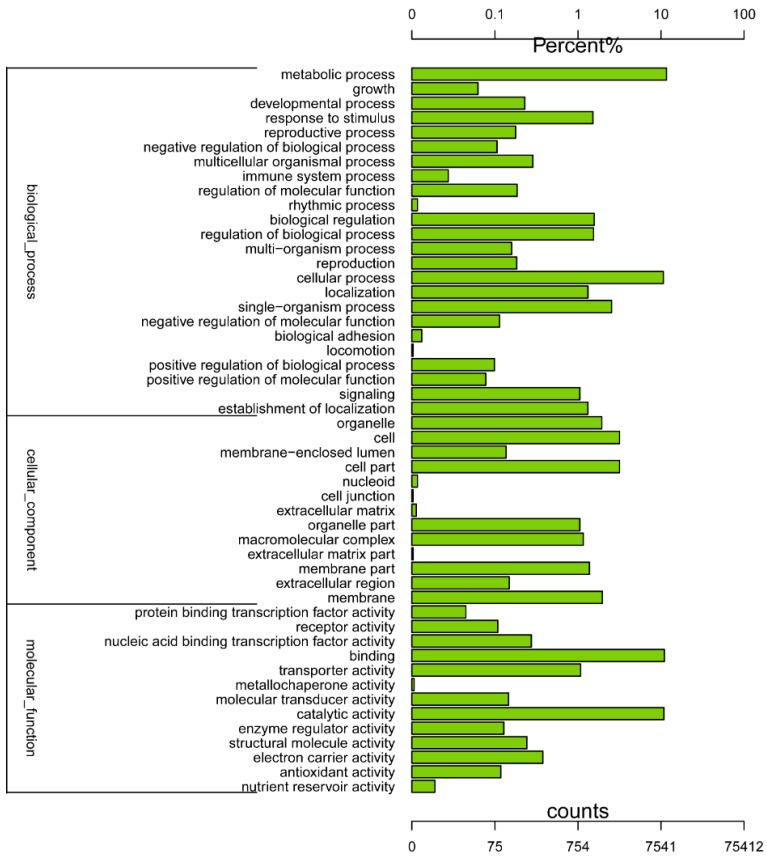
Gene Ontology functional classification. The classifications are shown in three main categories and fifty groups.

The GO was used to classify the functions of the predicted Chinese fir genes. Based on sequence homology, the 16,894 sequences were categorized into 50 functional groups (Figure 2). These unigenes were assigned into three main categories known as biological process, cellular component, and molecular function, in which terms the “Metabolic process”, “Cell part”, and “Binding” terms, respectively, were dominant. We also noticed a high percentage of genes from the “Cellular process”, “Membrane”, and “Catalytic activity” categories, but few from “Locomotion”, “Cell junction”, and “Metallochaperone activity” (Figure 2). The functional annotation of unigenes in GO showed that quite a few unigenes were categorized into multiple biological processes and molecular functions during drought stress. About 12,049 sequences were annotated as belonging to the “metabolic process” category, these could be the optional novel unigenes related to the secondary metabolism pathways in drought-stress tolerance (Figure 2).

KEGG is a database resource for understanding the high-level functions and utility of biological systems, at the cell, organism and ecosystem levels, from molecular-level information. Through sequence alignment with the KEGG database in BLASTx with an *E*-value cut-off of <10^−5^, of the 75,421 unigenes, 5887 (7.81%) had significant matches in the database and were assigned to 288 KEGG pathways (Table S3). The most represented pathways were “Metabolism” (2193 members, 37.25%), “Biosynthesis of secondary metabolites” (1211 members, 20.57%), and “Microbial metabolism in diverse environments” (438 members, 7.44%; Table S4). This annotation information would give an opportunity for further uncovering the molecular mechanism of Chinese fir short-term drought-stress response and long-term drought-stress tolerance.

### 2.3. Differently Expressed Genes during the Drought-Stress Treatments

**Figure 3:**
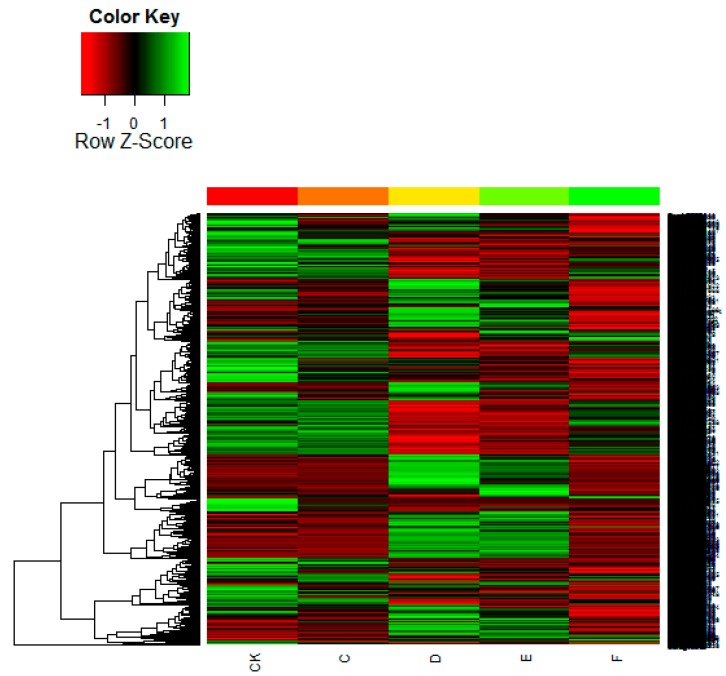
Changes in gene expression among drought-stress stages and re-watering. Heat-map of the total differentially expressed genes. Columns and rows in the heat maps represent samples and genes, respectively. Sample names are displayed below the heat maps. CK represents the control; C, D, and E represent the three drought treatment levels; F represents re-watering. Colour scale indicates fold changes in gene expression. A fold change of ≥1 is shown in green (increased transcript abundance), a fold change of  ≤ −1 is shown in red (decreased transcript abundance), and no change is indicated in black.

In order to investigate the genes involved in drought stress, we conducted an RNA-Seq experiment (well-watered plantlets [CK] as control, plantlets subjected to five days of drought [C], ten days of drought [D] and fifteen days of drought [E] as drought stress, and then one day post-rewatering [F] after fifteen days of drought as rewatering treatment) of Chinese fir and mapped the resulting reads to our reference transcriptome. To determine which of the 75,421 genes were differentially expressed among the five stages or treatments, we filtered with an FDR  ≤  0.05 and |log_2_ (ratio)|  ≥  1. The expression of 9326 DEGs was found to change significantly among the five treatments. Some genes were down-regulated from the CK to the drought treatments, then up-regulated after re-watering. On the other hand, expression of some genes increased gradually following extended water shortage, and then decreased upon rewatering. To identify genes showing a significant change in expression among the drought-stress treatments, the differentially expressed tags among the five stages were presented by heat-map (Figure 3).

We identified 10 DEGs groups by comparing the five libraries (*i.e.*, treatments CK, C, D, E, F; Figure S2). In the four pairwise groups compared with CK, the greatest number of DEGs was for the CK *vs.* D comparison, containing 1970 up-regulated and 2849 down-regulated genes. Regarding comparisons of the three drought treatments (*i.e.*, C *vs.* D, C *vs.* E, D *vs.* E), the number of DEGs was greatest for the C *vs.* D comparison (2209 up-regulated and 2235 down-regulated genes).

We also compared treatment F with the three drought-stress treatments. There were 4861 variable genes in the D *vs.* F comparison, which was the greatest of all of the treatment comparisons. This suggests that the differentiation of gene expression between F and D was greater than the other treatment comparisons, while the difference between C and CK was the smallest of the 10 comparisons. These results suggest that, in Chinese fir, transcript abundance changed dramatically at these key transitions among the drought-stress stages from 5 to 10 days. During this period, the drought response genes were induced and expressed, but genes expressed during the 0 to 5-days transition period should not be overlooked. Many important drought-stress response genes were up- and down-regulated during this period, which represents the earliest response of the plant to insufficient water. These findings suggest that our analysis was capable of identifying a large number of fir drought-response genes in Chinese fir, suitable for further investigation of their potential roles in providing drought tolerance.

### 2.4. SOM Cluster Analysis of Gene Expression Data

**Figure 4:**
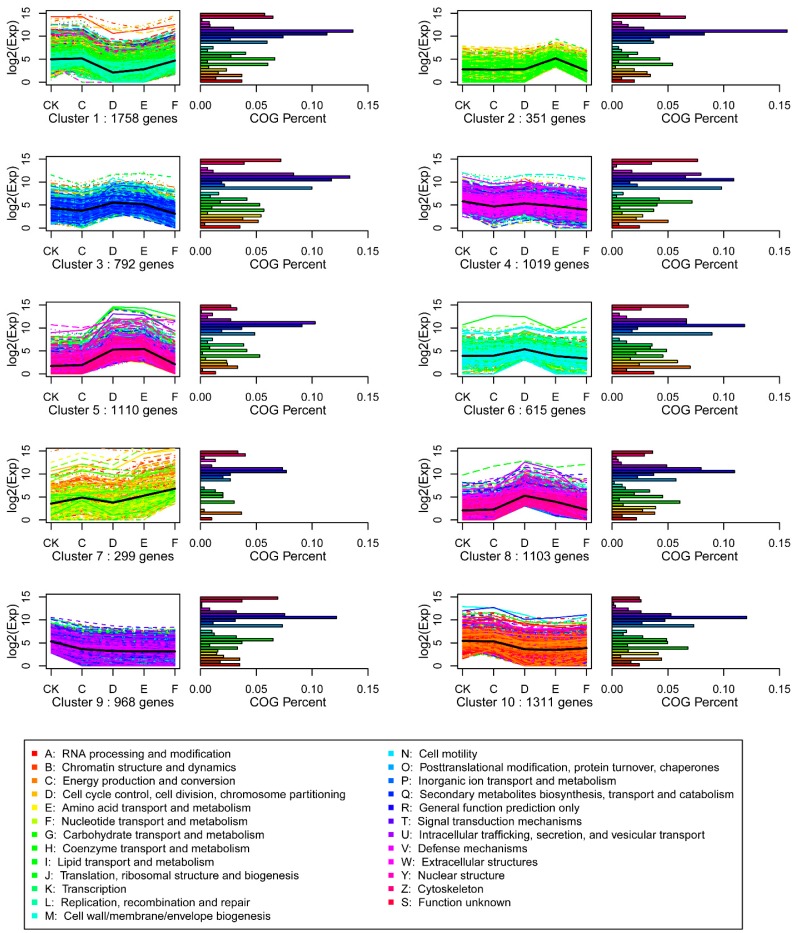
SOM cluster analysis of gene expression in the 10 different patterns. Clusters were obtained by the k-means method on the gene expression profiles of the 9326 modulated genes. The most abundant group is Clusters 1 and 10, with 1758 and 1311 genes whose expression shows a down-regulation in D relative to C. The second most abundant group is Clusters 5, 8, and 4, containing 1110, 1103, and 1019 genes, respectively, whose expression shows a positive slope from C to D.

To facilitate cluster analysis of gene expression data, expression profiles of the DEGs were determined by self-organising map (SOM) cluster analysis, based on the k-means method using Pearson’s correlation distance. The DEGs were divided into 10 groups, based on their expression modulation with analysis of GO and KEGG pathway enrichment, representing the number of profiles as indicated by figure of merit analysis (Figure 4). Clusters were obtained by the k-means method, using the gene expression profiles of the 9326 modulated genes. The most abundant groups were clusters 1 and 10, with 1758 and 1311 genes, respectively, whose expressions were negatively correlated during the transition from C to D. The second most abundant groups were clusters 5, 8, and 4, containing 1110, 1103, and 1019 genes, respectively, with a positive change in expression from C to D. There was an obvious peak in D of cluster 6 and E of cluster 2, which indicates that 615 gene expression values increased from C to D and 351 from D to E, respectively. Clusters 2, 3, and 5 comprised 2253 genes whose expression was down-regulated from E to F, indicating that re-watering affected their expression. Clusters 2, 3, 5, 6 and 8 comprised 1502 genes up-regulated from CK to C, D and E, but down-regulated in F. The pathways represented the “Signal transduction mechanisms; “Cytoskeleton”; “Secondary metabolites biosynthesis, transport and catabolism”; “General function prediction only”; “Carbohydrate transport and metabolism”; and “Amino acid transport and metabolism” genes. These results suggest that cells receive the drought signal, and respond by altering their cytoskeleton and increasing osmotic pressure to tolerate drought stress.

### 2.5. Gene Enrichment Analysis for DEGs among Five Libraries

GO terms are used to assess the biological significance of DEGs. The specific DEGs between the five libraries were analyzed for GO category enrichment (FDR ≤ 0.05), which involved 10 comparison groups (File S1). The 533 significant DEGs between primary drought and the CK treatment (CK *vs.* C), were categorized into 120 functional groups clustered into three main GO classification categories (Biological process, Cellular component, and Molecular function), which contained 49, 23, and 48 functional groups, respectively. Translation (GO: 0006412) with 64 genes was dominant in the Biological process category. Intracellular (GO: 0005622) consisted of 72 genes dominant in the main categories of the Cellular component. Structural constituent of the ribosome (GO: 0003735) included 63 genes dominant in the main category of Molecular function. Interestingly, these three dominant functional groups contained down-regulated genes.

Regarding GO enrichment of DEGs of moderate and severe drought treatments compared with the control (CK *vs.* D, CK *vs.* E), 2398 and 2231 genes were categorized into 60 and 61 functional groups, respectively. Similarly, the oxidation-reduction process (GO: 0055114) with 419 and 369 genes, and oxidoreductase activity (GO: 0016491) with 347 and 306 genes, dominated in the main categories of Biological process and Molecular function. In the main category of Cellular component, the dominant groups were chloroplast (GO: 0009507) with 103 genes, and intracellular (GO: 0005622) with 126 genes. For comparisons between the drought-stress treatments and rewatering, the response of DEGs to drought stress was enriched mainly in the following groups: oxidation-reduction process (GO: 0055114), metabolic process (GO: 0008152), and transport (GO: 0006810), which were clustered in the biological process, membrane (GO: 0016020), chloroplast (GO: 0009507), and nucleus (GO: 0005634), which were clustered in the Cellular component category; and oxidoreductase activity (GO: 0016491), catalytic activity (GO: 0003824), ATP binding (GO: 0005524), and metal ion binding (GO: 0046872), which were clustered in the Molecular function category. The genes in these expression clusters associated with different functional categories reflected the molecular and cellular events involved in the drought-stress response, tolerance, and defence in Chinese fir.

The DEGs among the five libraries were further analysed for KEGG pathway enrichment, according to various biological functions (FDR ≤ 0.05), using 10 comparison groups (File S2). Significantly enriched metabolic pathways and signal transduction pathways were identified. DEGs between mild drought stress and CK (CK *vs.* C) were enriched in 11 pathways, which was the fewest among the 10 comparison groups. The three main pathways in this group were biosynthesis of ribosome (ko03010), pyrimidine metabolism (ko00240), and oxidative phosphorylation (ko00190).

The second smallest group was the comparison of rewatering and CK (CK *vs.* F), the DEGs in which were enriched in 18 pathways. Metabolites (ko01110), ribosome (ko03010), and pyrimidine metabolism (ko00240) were the three main pathways. DEGs were enriched in more than 25 pathways in the remaining eight comparisons. The four main pathways were metabolic pathways (ko01100), biosynthesis of secondary metabolites (ko01110), microbial metabolism in diverse environments (ko01120), and carbon metabolism (ko01200). Other significantly enriched pathways with a large number of DEGs included several metabolic and signal transduction pathways related to drought, such as biosynthesis of amino acids (ko01230), glycolysis/gluconeogenesis (ko00010), citrate cycle (TCA cycle) (ko00020), starch and sucrose metabolism (ko00500), photosynthesis (ko00195), flavonoid biosynthesis (ko00941), glutathione metabolism (ko00480), carotenoid biosynthesis (ko00906), oxidative phosphorylation (ko00190), biosynthesis of amino acids (ko01230), and the calcium signalling pathway (ko04020).

### 2.6. The Changes of Number of DEGs Enriched in GO Oxidation-Reduction Process and KEGG Metabolic Pathways

Using the time-course design in this experiment, we detected 9326 DEGs in the 10 comparison groups (Figure S2). DEGs associated with oxidation-reduction processes represented the most abundant biological process category, with the exception of the CK *vs.* C comparison. Among the 10 comparison groups, 5-days mild drought (15 up- and 52 down-regulated genes) was the smallest group (Figure S3A). As drought stress progressed, 10-days moderate drought (CK *vs.* D) (169 up- and 250 down-regulated genes) was the dominant group (Figure S3A). The number of up-regulated genes declined substantially from severe drought to rewatering. Genes involved in signalling reactions or the transport of metabolites and electrons were associated with oxidation-reduction processes.

In this experiment, DEGs were categorized in KEGG metabolic pathways (ko01100), which was the largest comparison group and was consistent with our knowledge of GO oxidation-reduction processes (Figure S3B).

### 2.7. Responses of Important Differentially Expressed Genes to Drought Stress

**Figure 5:**
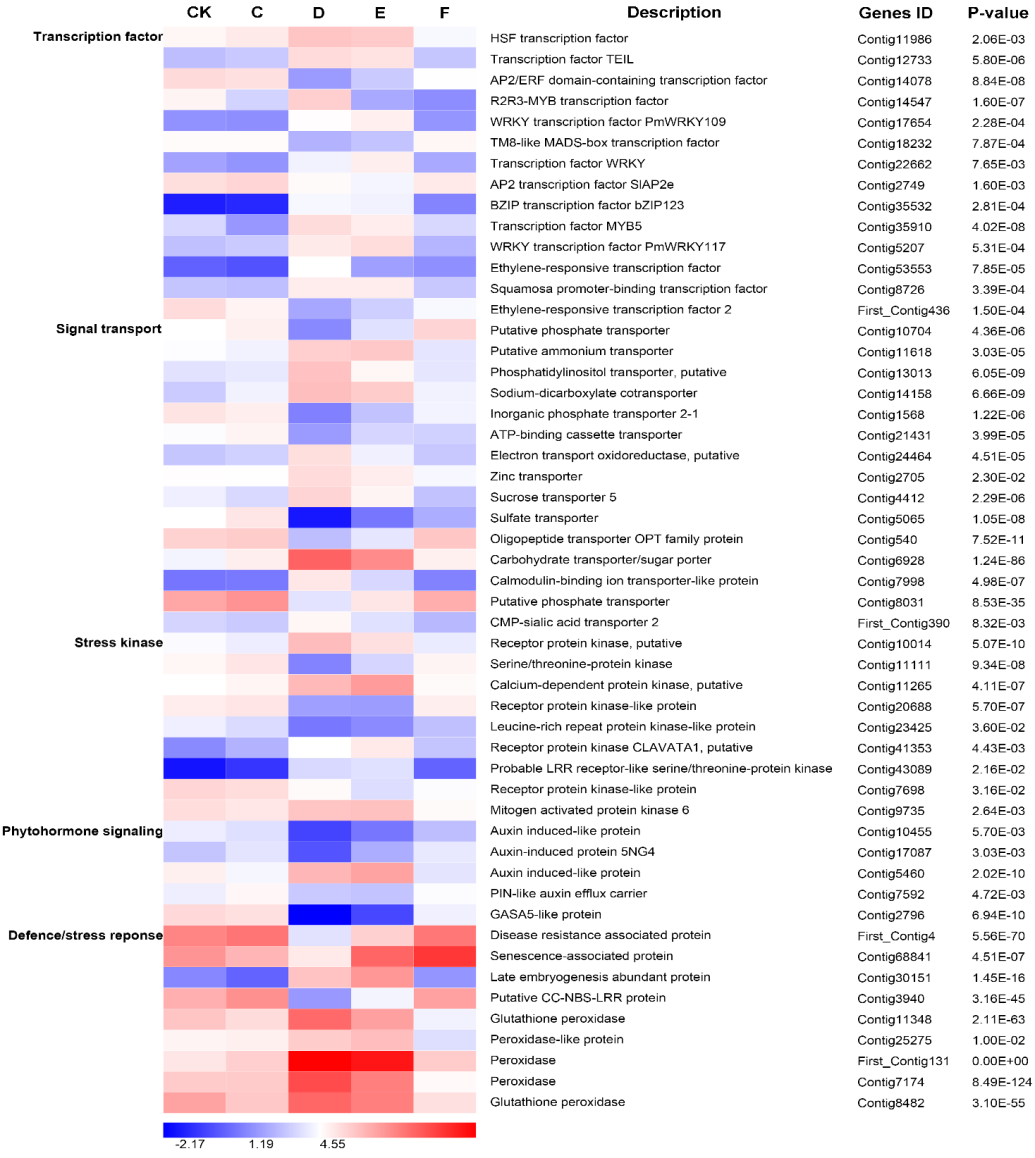
Heat-map of 52 differentially expressed genes. These genes were involved in transcription factor, signal transport, stress kinase, phytohormone signalling, and the defence/stress response, in the drought response and acclimation of Chinese fir. These genes were differentially expressed among the control 0 h (CK), drought treatments 5 day (C), 10 day (D) and 15 day (E), and re-watering (F). The bar represents the expression level of each gene (log2RPKM [number of reads per kilobase per million clean reads]) in the drought response and acclimation as indicated by blue/white/red rectangles. Low to high expression is indicated by a change in colour from blue to white to red. Complete information for each gene list can be found in Table S5.

For the 5-, 10-, and 15-days drought treatments, there were highly dynamic changes in transcript abundance (|log_2_ (ratio)| ≥ 2; Figure 5), included in unigenes sharing high sequence similarity with genes encoding TFs (HSF, TEIL, AP2/ERF, R2R3-MYB, PmWRKY109, TM8-like MADS-box, WRKY, SlAP2e, bZIP123, MYB5, PmWRKY117, ethylene-responsive, squamosa promoter-binding, and signal transport (putative phosphate transporter, putative ammonium transporter, phosphatidylinositol transporter putative, sodium-dicarboxylate cotransporter, inorganic phosphate transporter 2-1, ATP-binding cassette transporter, electron transport oxidoreductase putative, zinc transporter, sucrose transporter 5, sulphate transporter, oligopeptide transporter OPT family protein, carbohydrate transporter/sugar porter, calmodulin-binding ion transporter-like protein, putative phosphate transporter, CMP-sialic acid transporter 2)), stress kinase (receptor protein kinase, serine/threonine-protein kinase, calcium-dependent protein kinase putative, leucine-rich repeat protein kinase-like protein, receptor protein kinase CLAVATA1 putative, probable LRR receptor-like serine/threonine-protein kinase, mitogen-activated protein kinase 6), phytohormone signalling (auxin induced-like protein, auxin-induced protein 5NG4, PIN-like auxin efflux carrier, GASA5-like protein), and defence/stress response (disease resistance associated protein, senescence-associated protein, late embryogenesis abundant protein, putative CC-NBS-LRR protein, glutathione peroxidase, peroxidase-like protein, and peroxidase).

These changes in transcript expression patterns suggest differences in the response of Chinese fir to drought stress. For example, four TFs and one signal transport transcripts accumulated to the highest level at 15 days of water withholding, then decreased to the CK level after re-watering, indicating that some TFs reacted to the degree of drought stress (Figure 5; Table S5). Transcript levels of six TFs and eight signal transporters were higher at 10 days (moderate drought stress) than following the mild and severe drought-stress treatments, indicating that the moderate drought stress may induce changes in the expression of a greater number of genes than the mild and severe drought-stress treatments (Figure 5; Table S5).

The expression of a total of 16 genes showed different trends related to the duration of drought stress. The transcript level of each gene decreased with water deficiency and was lowest following moderate drought stress. These 16 genes comprised 3 TF-related genes (Contig14078, Contig18232 and First_Contig436), 6 signal-transport-related genes (Contig10704, Contig1568, Contig21431, Contig5065, Contig540 and Contig8031), 2 stress-kinase-related genes (Contig11111 and Contig23425), 3 phytohormone-signalling-related genes (Contig10455, Contig17087 and Contig2796), and 2 defence/stress-response genes (First_Contig4 and Contig3940) (Figure 5, Table S5). These results may contribute to identifying the transcriptional regulation signalling networks in response to drought stress in Chinese fir.

## 3. Discussion

### 3.1. Transcriptome Sequencing and Functional Annotation

In our study of the transcriptome of Chinese fir (2*n* = 22), approximately 37.2 GB of data were generated and assembled into 75,412 unigenes. Such a large number of reads (371,387,986 raw reads) produced considerably longer unigenes (average of 867 bp) than those in other research involving Chinese fir; e.g., two studies that performed cambial activity transcriptome sequencing and reported average unigene lengths of 497 and 505 bp [43,44]. This increased transcriptome coverage depth would help to enhance the accuracy of the sequencing and ensure the accurate assembly. What’s more, a great number of unigenes were assigned to various GO categories and COG classifications (Figure 1 and Figure 2), showing that various transcripts are associated with the drought-stress response. Furthermore, most representative unigenes were annotated to specific pathways, such as metabolic, biosynthesis of secondary metabolites, cell cycle, ribosome, biosynthesis of amino acids, pyrimidine metabolism, carbon metabolism, starch and sucrose metabolism pathways, using the KEGG database (Table S4). This led us to conclude that most of the genes identified were involved in the drought response and signalling regulation.

### 3.2. The Most Abundant Categories of DEGs Enriched in GO and KEGG Pathways

DEGs were categorized into GO oxidation-reduction processes (GO: 0055114), which typically represents the most abundant functional category. Peanut (*Arachis hypogaea* L.) dehydration for 30 min, using 30% PEG treatment, resulted in an identification of genes related to oxidation-reduction processes (17.14% of the total number of genes), which was the most enriched of the biological processes [45]. Long-term (5 weeks) water-deficit treatment of common bean (*Phaseolus vulgaris* L.) resulted in an identification of genes allocated to oxidation-reduction pathways, which was one of the most overrepresented biological process GO categories [46]. Since the DEGs categorized into oxidation-reduction processes were the most significant category of biological process for both short- or long-term dehydration, it would be interesting to evaluate the effect on the DEGs of drought-stress intensity. In our experiment, we got similar results compared with the previous studies. This severe water stress resulting in more DEGs in the oxidation-reduction processes indicates that Chinese fir plantlets require a greater signalling transport capacity to respond to severe water deficit.

In previous studies, 593 (21.93%) genes were categorized into metabolic pathways among the 2704 differently expressed Black Cottonwood (*Populus trichocarpa* [Torr. & A. Gray]) genes identified under drought-stress conditions [47]. In KEGG analysis of drought stress in two *Paulownia fortune* genotypes, the greatest number of DEGs was related to metabolic pathways under a 12-days drought-stress treatment [48]. In a comparative analysis of drought-responsive transcriptome in Indica rice genotypes with differing drought tolerance, DEGs were categorized into metabolic pathways and a significant up-regulation of the a-linoleic acid metabolic pathway was detected in the stress-tolerant genotype N22 [49]. These studies were consistent with our experimental data regarding categorising DEGs into metabolic pathways. In contrast with previous studies, however, we also monitored the quantitative variation of DEGs enriched in this category. Interestingly, variation in the number of DEGs categorized into KEGG metabolic pathways was similar to the changes in oxidation-reduction processes among the 10 treatment groups (Figure S3B). This suggests that Chinese fir plantlets require stronger protective mechanisms to respond and adapt to severe water deficit.

### 3.3. Important TF Genes Involved in Drought Stress and Re-Watering

Reports have indicated that numerous TF families—such as MYB, DREB, bZIP, and WRKY—are directly or indirectly involved in the regulation of the plant response to drought stress [10]. Many IF genes have been previously identified as key regulators of drought responses in plants.

The MYB TFs comprise one of the largest gene families in plants. Many plant MYB genes involved in the response to drought stress have been identified and functionally characterized [17]. The overexpression of *AtMYB15* results in enhanced drought tolerance and sensitivity to abscisic acid (ABA) [50]. Detailed characterisation of *Arabidopsis* transgenic plants that overexpress the *TaMYB30-B* gene from wheat may facilitate improvement of drought-stress tolerance during the germination and plantlet stages [51]. Also from wheat, over-expression of *TaMYB33* in *Arabidopsis* markedly enhanced its tolerance to drought and NaCl stresses [52]. Seven DEGs encoding the MYB TF were detected in this study (File S3). Four DEGs showed significant up-regulation in response to drought stress, and down-regulation following rewatering. Interestingly, the other three DEGs were negatively expressed compared to the aforementioned four DEGs, indicating that these three genes were negatively related to drought tolerance.

The role of WRKY TFs in abiotic stress signalling networks is becoming apparent and proteomic reports have identified WRKY proteins that are up-regulated by drought stress [16]. Previous studies have shown that overexpression of WRKY genes (e.g., ABA-inducible *OsWRKY45* gene, *OsWRKY11*, and *OsWRKY08*) enhanced plant tolerance to drought [53]. A WRKY up-regulated gene that was a candidate TF for regulation of the drought response was identified in pine roots under drought-stress conditions [54]. Eight DEGs in response to drought stress were identified in our study, notably those encoding the WRKY TFs Congtig17654 and Contig5207, which showed highly positive changes in expression in response to drought stress (File S3).

AP2/ERF family is a large group of plant-specific transcription factors that includes four major subfamilies named the AP2, RAV, ERF, and DREB based on their sequence similarities and a number of AP2/ERF domains [55]. Previous research has demonstrated that overexpression of AP2/ERF genes enhances resistance to biotic and abiotic stresses, such as gene *GmERF3*, which might play dual roles in cross-talk between the biotic and abiotic stress signal pathways in plants [56]. In the present study, eighteen putative AP2/ERF genes showed different changes in response to drought stress (File S3). Genes showing highly positive (such as Contig53553) or negative (such as Contig14078, Contig2749, and First_Contig436) changes may play an important role in response to drought.

Previous studies have demonstrated that basic leucine zipper (bZIP) is a large transcription factor family in plants, and the members of bZIP have diverse roles, especially in plant stress-response and hormone signal transduction [57,58]. Heterologous overexpression of *ZmbZIP72*, from maize, improved transgenic Arabidopsis’ drought and partial salt tolerance, which was determined by physiological analyses, such as leaf water loss, electrolyte leakage, proline content, and survival rate under stress [59]. In our experiment, two putative bZIP genes (Contig35532 and Contig12547) showed significant up-regulation in response to drought stress, and down-regulation following rewatering.

Additional TF genes, such as Contig11986 (putative HSF gene) and Contig18232 (putative MADS gene), were also identified in Chinese fir plantlets exposed to drought stress and drought recovery, with expression trends similar to those of the two WRKY TF genes (File S3). These genes may have potential usefulness in improving the stress resistance of Chinese fir.

### 3.4. Differentially Expressed Genes Related to Phytohormone Signaling under Drought Stress and Re-Watering

As a well-known plant phytohormone, auxin is an important regulator involved in multiple plant growth processes and stress responses [60]. Generally, subjected to drought stress, plants would reduce the internal auxin concentration by changing the genes’ transcript level which were involved in the auxin biosynthesis and signaling pathways [61]. Auxin homeostasis and signaling are often modulated through the regulation of YUCCA and Aux/IAA genes [62]. For example, the rice Aux/IAA gene *OsIAA6* is highly induced by drought stress and its overexpression in transgenic rice improved drought tolerance [63]. Auxin is actively transported through influx and efflux carriers, which are positioned asymmetrically on the plasma membrane. The distinct subcellular localization of PIN (PIN-FORMED) auxin efflux carriers determines the overall direction of auxin flux [64]. Previous study in rice found that *OsPIN3t* played a key role in shoot and root development, and was involved in drought stress responses [65]. In the current study, some putative auxin induced genes and a PIN-like auxin efflux carrier gene had been detected and showed a down regulation in drought stress (Figure 5, File S4). These down-regulated genes may have a relationship with the endogenous auxin concentration according to the levels of dehydration.

Abscisic acid (ABA) acts as a central regulator in the response and adaptation to drought conditions. The various physiological reactions regulated by ABA—such as stomatal closure, changes in gene expression and accumulation of osmoprotectants—have been characterized at the molecular level [66,67,68]. The molecular mechanisms of ABA synthesis, transport, and signalling have received substantial attention and are now reasonably well understood [69]. In our study, a series of ABA synthesis-related enzymes and transporters were identified in plants exposed to drought stress.

Recent studies have shown that, upon perception of a signal, ABA is synthesized primarily in vascular tissues and then exported to other cells in the plant [70]. The gene 9-cis-epoxycarotenoid dioxygenase (NCED) catalyzes the first step and is the key enzyme in ABA biosynthesis. In transgenic *Arabidopsis* and tobacco (*Nicotiana tobacum* L.), overexpression of *AtNCED3* and *PvNCED1*, respectively, caused an increase in endogenous ABA levels and enhanced drought-stress tolerance [71,72]. In grapevine (*Vitis* spp.) at various irrigation levels, the expression of genes associated with ABA synthesis (*NCED1* and *NCED2*) in roots was correlated with ABA abundance in the roots, xylem sap, and leaves [73]. In the present study, the transcript abundance of two genes thought to encode NCED was significantly related to drought intensity (File S3). For Contig3270, approximate 3-, 74-, and 104-fold up-regulations were found in mild, moderate, and severe drought-stress treatments, respectively, compared with the control. The expression of this gene was 249-fold down-regulated during drought recovery compared with the severe drought. These observations are consistent with the regulatory role of NCEDs in water stress in angiosperms and suggest NCED to exert a similar function in Chinese fir.

ABA responses require translocation from ABA-producing cells via intercellular transport to allow a rapid distribution into neighbouring tissues. Recently, cell-to-cell ABA transport was shown to be mediated by two plasma membrane-bound ATP-binding cassette (ABC) transporters, ABCG25 and ABCG40 [74,75]. The ABCG25 and ABCG40 transporters have been reported to play a role in mediating ABA export from inside to outside the cell and import from outside to inside the cell, respectively. The *ABCG25* gene is expressed mainly in vascular tissues and is induced by ABA and drought stress, whereas *ABCG40* is expressed in guard cells [74,75,76]. These findings suggest an ABA transport model in leaves, where ABA is synthesized in vascular tissues in response to drought stress. In our Chinese fir plantlet transcriptome, 29 ATP-binding cassette (ABC) genes were detected in response to water stress, of which four ABC genes showed differential expression in the presence of drought stress (File S3). Among these four DEGs, a strongly up-regulated gene Contig9655, which exhibited high homology with the *Arabidopsis ABCG40* gene, was identified as a potential Chinese fir ABA import ABC regulator. Therefore, Contig9655, as an ABC transporter gene, may act in ABA transport in Chinese fir plantlets upon exposure to drought stress.

### 3.5. The Other Candidate Functional and Regulatory Genes Involved in Drought Stress and Re-Watering

Some other candidate functional and regulatory genes have been detected in changes of transcript levels in response to drought stress. These genes were mainly categorized into three categories *i.e.*, signal transport, stress kinase, and defence/stress reponse.

Receptor-like kinases (RLKs), which contains Ser/Thr kinase, convey signal to their target proteins in the cytoplasm by catalytic processes of protein kinase activity. Transcriptome analyses showed that a number of *RLK* genes were up-regulated by biotic stresses and RLKs could play an important role in optimizing plant response to drought stress [77]. For example, overexpression of a putative RLK gene *OsSIK1* in transgenic rice showed higher tolerance to salt and drought stresses than control plants [78]. Mitogen-activated protein kinases (MAPK) pathways are known to be activated by numerous abiotic stresses such as cold, salt, heat, drought, ozone, or heavy metal intoxication [79]. MAPK, MAPKK (MAPK kinase), and MAPKKK (MAPKK kinase) are functionally linked and operate as an important network for regulating signals from the upstream receptors to the downstream cellular effectors. Previous study had demonstrated that *PtrMAPK* acted as a positive regulator in dehydration/drought stress responses [79]. Calcium-Dependent Protein Kinases (CDPKs) are directly activated by the binding of Ca^2+^ to the calmodulin-like domain, and activated CDPKs regulate downstream components of calcium signaling. In rice, *OsCPK4* overexpressor plants exhibited stronger water-holding capability under drought or salt stress [80]. In this study, the DEGs encoding putative RLKs, LRR-RLK, MAPK, and CDPKs may provide unique opportunities for increasing drought resistance in Chinese fir.

Secondary signaling molecules such as protein kinases and phosphatases (serine/threonine phosphatases), phospholipids like phosphoinositides, Ca^2+^, nitric oxide, cAMP and sugars, play an important role in signal transduction [81]. In the lipid signaling events, phospholipid signaling had been well studied as a second messenger system because of the lipid molecules’ dual function of a structural role and a signal-transducing property. In eukaryotic cells, the phosphatidylinositol synthesis is catalyzed by phosphatidylinositol synthase (PIS). Previous study has demonstrated that the overexpression of *ZmPIS* in tobacco plants changed membrane lipids composition and improved drought stress tolerance [82]. Iron is essential for the survival and proliferation of all plants. Transporters are thought to function inside plant to facilitate internal iron transport. Previous study showed that the KUP6 (K^+^ uptake transporter 6) subfamily transporters acted as key factors in osmotic adjustment by balancing potassium homeostasis in cell growth and drought stress responses [83]. The other putative transporter genes were induced in drought stress conditions, such as the genes encoding oligopeptide transporters and sugar transporters [84,85].

Functional genes play a role in protecting plant from damage produced by reactive oxygen species (ROS), such as peroxidase, superoxide dismutase, and glutathione S-transferase (GST). These genes encode proteins that contribute directly to cellular stress tolerance, such as LEA (late embryogenesis abundant) proteins, molecular chaperones, enzymes for detoxification of reactive oxygen species, and those for the biosynthesis of sugars or proline, which are important as osmolytes and/or protectants. Late embryogenesis abundant (LEA) proteins act as key roles in plant desiccation tolerance. In *Arabidopsis thaliana*, most of 51 LEA protein encoding genes had abscisic acid response (ABRE) and/or low temperature response (LTRE) elements in their promoters and many genes containing the respective promoter elements were induced by abscisic acid, cold, or drought [86]. In this study, a putative LEA gene (Contig30151) showed high up-regulation in drought stress. Previous research had showed that overexpressing *IbLEA14* in sweet potato transgenic calli enhanced tolerance to drought and salt stress [87]. Apart from LEA, changes of the transcript levels of the other putative functional genes, encoding senescence-associated protein, glutathione peroxidase, and peroxidase-like protein, had been also detected in Chinese fir drought stress. These DEGs may play an important role in Chinese fir drought response and tolerance.

## 4. Experimental Section

### 4.1. Ethics Statement

Chinese fir is widely distributed in southern China and is not listed as endangered or protected species. No specific permissions are required for sample collection in the Guangdong province, and that the field studies did not involve any endangered or protected species.

### 4.2. Plant Growth, Drought Stress, and Sampling

The elite Chinese fir sample “GZ7” was collected from the Guangdong Province in southern China. The annual plant material was propagated from tissue culture of adult mother plants. The 15–20-cm-tall plantlets were selected and planted in pots (18-cm diameter at the top, 13 cm at the bottom, and 17-cm height) filled with a mixture of peat soil, washed sand, vermiculite, and perlite (3:1:1:1 in volume). Clones were grown in a controlled-environment growth chamber at the Beijing Forestry University. The chambers were set at 25 °C day/20 °C night, with a 16-h day/8-h night photo cycle and 70% relative humidity. Photon flux density at the soil level was 200–250 μM·m^−2^·s^−1^, supplied mainly with cool light. A total of 25 plantlets were divided into five equal groups: five well-watered plantlets as control (CK), three levels of drought stress treatments (five plantlets of each level), and one rewatering treatment.

The CK plants were watered every 3 days, in the early morning, and the average soil moisture was 43.04%. Plantlets would undergo three levels of drought stress from 10% soil moisture. Water-deficit stress was imposed by withholding water for 5, 10, and 15 days, corresponding to mild, moderate, and severe, levels of drought stress, respectively. The average soil moisture of mild, moderate and severe treatments were 4.39%, 2.66%, and 2.23%, respectively. The three levels of drought stress were abbreviated as C, D, and E, respectively. The rewatering treatment involved 24-h post-rewatering plants that had experienced 15 days of drought stress, and was abbreviated as F. The average soil moisture of rewatered plantlets was 43.18%.

Three plantlets were randomly selected from every treatment. Fifteen segments of needles, approximately 4 cm long, were collected 5 cm from the plantlet tip, then combined as a bulk sample. Each bulked sample was collected at approximately 10:00 h, under similar environmental conditions. The samples were immediately frozen in liquid nitrogen, then stored at −80 °C for subsequent RNA extraction at the completion of sampling. Total RNA in the needles was extracted using an RNAisomate RNA Easyspin Isolation System (Aidlab Biotech, Beijing, China) according to the manufacturer’s instructions. The quality of RNA was verified with a 2100 Bioanalyzer (Agilent Technologies, Santa Clara, CA, USA) and RNA integrity number (RIN > 8.0). To prepare cDNA, we used 60-μg pooled RNA from each sample.

### 4.3. cDNA Library Preparation and Transcriptome Sequencing

Illumina sequencing was conducted using the Solexa mRNA-Seq platform according to the manufacturer’s instructions (Illumina, San Diego, CA, USA). Briefly, we used magnetic beads with oligo(dT) to isolate poly(A) mRNA after isolating total RNA from Chinese fir leaves. Double-stranded cDNA was synthesized using appropriate buffers, dNTPs, RNase H, and DNA polymerase I (ShoBiotechnology Corporation, Shanghai, China). The fragments were purified with a QiaQuick PCR extraction kit (Qiagen, Hilden, Germany) and eluted with elution buffer for end repair and by addition of poly(A). For PCR amplification, we selected suitable fragments as templates, based on the results of agarose gel electrophoresis. The library was sequenced paired end 100 nt multiplex using an Illumina HiSeq™ 2000. Because raw reads produced from sequencing machines contain low-quality reads that negatively affect subsequent bioinformatics analyses, we discarded reads with adapters. We also discarded those with more than 5% unknown nucleotides, and those of low quality (≤50% of the bases with a quality score [Q] ≤ 20), using an in-house Perl script. The average proportion of clean reads in each sample was 88.6%–94.3%.

### 4.4. Analysis of Illumina Transcriptome Sequencing Results

*De novo* assembly was performed using the scaffolding contig methods of the CLC Genomics Workbench (version 6.0.4) with word-size of 45 and a minimum contig length of ≥300 [88,89]. The assembled *De novo* sequences were designated as primary unigenes. After assessing the various K-mer sizes, we found that 29-mer was suitable for assembling, so this size class was selected to construct the de Bruijn graph. Primary unigenes from UniGene of five samples were assembled using CAP3 EST, yielding final unigenes. Assembled final unigenes were used for BLASTx searches (*E*-value < 1 × 10^−5^) against the Swiss-Prot protein database (date: April 2013) [90], which is a high quality annotated and non-redundant protein annotation database (including 24,889,084 proteins). To functionally annotate sequences, we used the Blast2GO software [91] to assign gene ontology (GO) terms (date: April 2013) (Available online: http://www.geneontology.org). Also, to predict and classify possible functions, 15,662 unigene sequences were aligned to 25 Clusters of Orthologous Groups (COGs) in the COG database (date: April 2013) (Available online: http://www.ncbi.nlm.nih.gov/COG). Kyoto Encyclopedia of Genes and Genomes Pathway (date: April 2013) (KEGG; Available online: http://www.genome.jp/kegg) annotations were carried out according to the KEGG database, using BLASTx (*E*-value threshold 10^−5^).

### 4.5. Bioinformatics for Functional Annotation of Differentially Expressed Genes

The clean read expression distribution was used to evaluate the data for normality. All clean reads were mapped to our transcriptome reference sequences using SOAPaligner/soap2, and mismatches as small as 1 bp were considered. The number of unambiguous clean reads for each gene was calculated and then normalized to the number of reads per kilobase per million clean reads (RPKM), uniquely aligning within each sample.

A rigorous algorithm to identify differentially expressed genes was developed, based on the method of Audic and Claverie [92]. The false discovery rate (FDR) was used to determine the threshold of the *p*-value in multiple tests and analyses. We used an FDR of <0.05 and the absolute value of log2 (ratio) ≥2, as thresholds to define significantly differential gene expression. For further analyses, we used an additional criterion, which involved using only DEGs with a minimum fourfold change in expression.

### 4.6. Gene Ontology Functional and KEGG Pathway Enrichment Analysis for DEGs

Gene Ontology (GO) analysis provides a common descriptive framework and functional annotation and classification for analysing the DEGs. All DEGs were examined for each term of the GO database (Available online: http://www.geneontology.org/), and the gene numbers for each GO term were recorded. To identify significantly enriched GO terms in DEGs, a hypergeometric test was used and performed as described previously [93]. The calculated p-value was subjected to Bonferroni correction, with a corrected *p*-value (FDR) ≤0.05 as a threshold. GO terms fulfilling this criterion were defined as significantly enriched in DEGs, within the context of the whole transcriptome background. This analysis identified the main biological functions associated with the DEGs. GO functional enrichment analysis also integrated the clustering analysis of expression patterns to easily determine the expression patterns of DEGs annotated to a given GO term.

Pathway enrichment analysis was based on the KEGG database (Available online: http://www.genome.jp/kegg/) and facilitated retrieval of significantly enriched pathways associated with DEGs, within the context of the whole transcriptome background. The formula for calculating the *p*-value was similar to that used in GO analysis. The calculated *p*-value was also subjected to Bonferroni correction, with a corrected *p*-value (FDR) ≤0.05 as a threshold.

## Supplementary Materials

The authors confirm that all data underlying the findings are fully available without restriction. All relevant data are available from the NCBI SRA database (accession number SRP052290) and within Supporting Information files. The details can be found at http://www.mdpi.com/1422-0067/16/07/15194/s1.
