# Supplementary material for: Global Reprogramming of Transcription in Chinese Fir (Cunninghamia lanceolata) during Progressive Drought Stress and after Rewatering
Source: Int J Mol Sci. 2015 Jul 6;16(7):15194–219. doi: 10.3390/ijms160715194 (PMC4519895; doi:10.3390/ijms160715194)
Supplement: Supplementary file 1 [file ijms-16-15194-s001.zip › ijms-83291-supplementary information/ijms-83291-Supplementary Information.pdf]

Supplementary Information

Table S1. Overview of the sequencing and assembly.

Sample ID	Raw Bases (G)	Q20 Value (%)	Raw Reads	Quality Trimmed	Adaptor Trimmed	Number Clean Reads	rRNA Trimmed	Clean Ratio
CK	5.7	97.5	56,930,846	56,407,568	55,809,822	54,764,610	53,697,348	94.30%
C	8.7	97.5	86,707,968	85,720,076	84,841,030	83,117,122	76,853,198	88.60%
D	6.6	97.5	65,985,062	65,338,791	64,654,791	63,442,220	60,907,814	92.30%
E	8.8	96.6	87,715,970	87,600,546	87,513,980	87,322,067	79,741,790	90.91%
F	7.4	97.1	74,048,140	73,936,606	73,867,663	73,699,083	67,316,490	90.90%

Q20 = bases of Q ≥ 20/all bases of sequencing; Clean ratio = rRNA trimmed/raw reads.

Table S2. Summary of the Chinese fir (*Cunninghamia lanceolata*) transcriptome.

Statistics	Counts	Total Length (bp)	N25 (bp)	N50 (bp)	N75 (bp)	Average Length	Longest (bp)	N%	GC%
contigs	120,924	74,108,516	1946	850	387	613	19,035	0.3	39.4
Primary UniGene	77,229	65,646,361	2396	1252	538	850	26,392	0.3	39.4
Final UniGene	75,412	65,290,699	2430	1289	552	867	26,392	0.4	39.4

Table S3. Annotation of unigene sequences in Chinese fir (*Cunninghamia lanceolata*).

Sequence Database	Number of Annotated Unigene Sequences	Percentage of Annotated Unigene Sequences
Total unigenes	75,421	100
Swiss-Prot	27,634	36.64
COG	15,662	20.77
GO	16,894	22.40
KEGG	5887	7.81

Swiss-Prot, Swiss-Prot protein database; COG, clusters of orthologous groups; GO, gene ontology; KEGG, Kyoto Encyclopedia of Genes and Genomes Pathway.

Table S4. Top 50 categories of Chinese fir unigenes enriched into KEGG pathways.

KEGG Categories	Mapped-KO	Unigene-NUM	Rate of No.	Pathway-ID
Metabolic pathways	823	2193	37.25	ko01100
Biosynthesis of secondary metabolites	345	1211	20.57	ko01110
Microbial metabolism in diverse environments	131	438	7.44	ko01120
Cell cycle	60	324	5.50	ko04110
Ribosome	113	280	4.76	ko03010
Biosynthesis of amino acids	102	272	4.62	ko01230
Pyrimidine metabolism	73	272	4.62	ko00240
Carbon metabolism	88	265	4.50	ko01200
Spliceosome	98	239	4.06	ko03040
Starch and sucrose metabolism	35	219	3.72	ko00500
Plant-pathogen interaction	34	206	3.50	ko04626
Carbon fixation in photosynthetic organisms	26	204	3.47	ko00710
Plant hormone signal transduction	37	200	3.40	ko04075

Table S4. *Cont.*

KEGG Categories	Mapped-KO	Unigene-NUM	Rate of No.	Pathway-ID
Protein processing in endoplasmic reticulum	75	196	3.33	ko04141
RNA transport	93	195	3.31	ko03013
Epstein-Barr virus infection	63	185	3.14	ko05169
Phenylpropanoid biosynthesis	15	184	3.13	ko00940
Purine metabolism	86	174	2.96	ko00230
Herpes simplex infection	30	163	2.77	ko05168
Glycolysis/Gluconeogenesis	34	156	2.65	ko00010
Viral carcinogenesis	42	155	2.63	ko05203
HTLV-I infection	48	152	2.58	ko05166
Oxidative phosphorylation	77	147	2.50	ko00190
Endocytosis	39	139	2.36	ko04144
Galactose metabolism	15	135	2.29	ko00052
Ubiquitin mediated proteolysis	61	133	2.26	ko04120
mRNA surveillance pathway	48	130	2.21	ko03015
Phenylalanine metabolism	16	129	2.19	ko00360
Amino sugar and nucleotide sugar metabolism	42	127	2.16	ko00520
Citrate cycle (TCA cycle)	23	124	2.11	ko00020
ErbB signaling pathway	8	121	2.06	ko04012
RNA degradation	49	120	2.04	ko03018
Cell cycle—yeast	54	119	2.02	ko04111
Influenza A	20	119	2.02	ko05164
Nitrogen metabolism	14	117	1.99	ko00910
MicroRNAs in cancer	20	114	1.94	ko05206
Oocyte meiosis	32	114	1.94	ko04114
Phagosome	31	110	1.87	ko04145
Alcoholism	15	107	1.82	ko05034
Apoptosis	8	106	1.80	ko04210
Pyruvate metabolism	31	105	1.78	ko00620
Neurotrophin signaling pathway	18	104	1.77	ko04722
Stilbenoid, diarylheptanoid and gingerol biosynthesis	5	101	1.72	ko00945
Glutathione metabolism	17	100	1.70	ko00480
Peroxisome	39	97	1.65	ko04146
Cysteine and methionine metabolism	32	97	1.65	ko00270
Histidine metabolism	12	97	1.65	ko00340
Non-alcoholic fatty liver disease (NAFLD)	42	94	1.60	ko04932
Arginine and proline metabolism	41	92	1.56	ko00330
Insulin signaling pathway	20	92	1.56	ko04910

Table S5. Transcript expression level of 52 differentially expressed genes.

Category Annotation	Genes ID	Unigene_ Length	Description	CK_RPKM	C_RPKM	D_RPKM	E_RPKM	F_RPKM	*p*-Value
Transcription factor	Contig11986	2123	HSF transcription factor	21.60193	26.90144	56.285	51.08365	15.44308404	2.06×10^{-3}
Transcription factor	Contig12733	2920	Transcription factor TEIL	4.966853	6.165395	36.42678	31.83624	5.776346314	5.80×10^{-6}
Transcription factor	Contig14078	626	AP2/ERF domain-containing transcription factor	37.38483	32.90566	2.497829	6.278978	18.49667789	8.84×10^{-8}
Transcription factor	Contig14547	1852	R2R3-MYB transcription factor	22.15254	7.506262	45.96736	3.297967	1.870712121	1.60×10^{-7}
Transcription factor	Contig17654	3849	WRKY transcription factor PmWRKY109	2.026752	1.923602	18.57196	24.45211	2.193448397	2.28×10^{-4}
Transcription factor	Contig18232	799	TM8-like MADS-box transcription factor	19.33431	19.30203	3.938155	5.468087	21.47519845	7.87×10^{-4}
Transcription factor	Contig22662	1850	Transcription factor WRKY	2.898266	2.16713	14.04512	25.18801	3.262500651	7.65×10^{-3}
Transcription factor	Contig2749	594	AP2 transcription factor SlAP2e	34.41541	40.8976	19.9239	14.71154	27.54689684	1.60×10^{-3}
Transcription factor	Contig35532	1399	BZIP transcription factor bZIP123	0.204195	0.252538	15.33022	13.9227	1.577110044	2.81×10^{-4}
Transcription factor	Contig35910	360	Transcription factor MYB5	8.17942	2.304132	35.33743	25.89579	8.306837005	4.02×10^{-8}
Transcription factor	Contig5207	2565	WRKY transcription factor PmWRKY117	5.200218	6.28808	27.15383	35.22272	4.030788688	5.31×10^{-4}
Transcription factor	Contig53553	945	Ethylene-responsive transcription factor	0.790619	0.585176	17.87427	2.659547	1.96817044	7.85×10^{-5}
Transcription factor	Contig8726	4407	Squamosa promoter-binding transcription factor	5.649461	5.061039	26.19012	26.33616	6.107136804	3.39×10^{-4}
Transcription factor	First_Contig436	1485	Ethylene-responsive transcription factor 2	36.37271	22.62239	3.17187	6.956711	15.63134295	1.50×10^{-4}
Signal transport	Contig10704	501	Putative phosphate transporter	17.93931	23.7925	1.733911	9.653866	42.11045895	4.36×10^{-6}
Signal transport	Contig11618	1385	Putative ammonium transporter	17.23059	13.97452	46.42767	53.28912	10.71689494	3.03×10^{-5}
Signal transport	Contig13013	2425	Phosphatidylinositol transporter, putative	10.21251	11.29421	60.89781	21.4089	10.88055703	6.05×10^{-9}
Signal transport	Contig14158	2533	Sodium-dicarboxylate cotransporter	6.419729	13.72958	64.63442	49.126	13.70648502	6.66×10^{-9}
Signal transport	Contig1568	2563	Inorganic phosphate transporter 2-1	30.5226	25.26185	1.48378	5.341976	14.16501359	1.22×10^{-6}
Signal transport	Contig21431	477	ATP-binding cassette transporter	16.81491	22.09133	2.428202	7.872737	7.224998592	3.99×10^{-5}
Signal transport	Contig24464	1388	Electron transport oxidoreductase, putative	5.84194	7.237764	34.42213	13.31717	6.016866194	4.51×10^{-5}
Signal transport	Contig2705	1889	Zinc transporter	18.53122	18.45908	36.39083	26.24599	15.39653775	2.30×10^{-2}
Signal transport	Contig4412	2521	Sucrose transporter 5	13.04004	8.451227	42.35287	22.10644	5.403088707	2.29×10^{-6}
Signal transport	Contig5065	2816	Sulfate transporter	17.82312	29.30896	0.178235	1.193455	3.53552843	1.05×10^{-8}
Signal transport	Contig540	3202	Oligopeptide transporter OPT family protein	43.90794	49.34479	4.991844	10.80616	55.25594942	7.52×10^{-11}
Signal transport	Contig6928	685	Carbohydrate transporter/sugar porter	14.37168	24.98105	402.063	176.2831	24.33044017	1.24×10^{-86}
Signal transport	Contig7998	3520	Calmodulin-binding ion transporter-like protein	1.179884	1.252436	28.63275	8.086438	1.517816601	4.98×10^{-7}

Table S5. *Cont.*

Category Annotation	Genes ID	Unigene_ Length	Description	CK_RPKM	C_RPKM	D_RPKM	E_RPKM	F_RPKM	*p*-Value
Signal transport	Contig8031	365	Putative phosphate transporter	105.0565	154.8293	10.3661	29.46429	90.77294056	8.53×10^{-5}
Signal transport	First_Contig390	2623	CMP-sialic acid transporter 2	7.749321	6.336437	21.40169	9.804498	4.463043082	8.32×10^{-3}
Stress kinase	Contig10014	2717	Receptor protein kinase, putative	16.41015	12.54538	67.8809	33.28448	12.08699705	5.07×10^{-10}
Stress kinase	Contig11111	3098	Serine/threonine-protein kinase	21.20141	29.97802	1.532871	7.801266	22.2133891	9.34×10^{-8}
Stress kinase	Contig11265	2794	Calcium-dependent protein kinase, putative	17.49943	21.88678	71.3095	131.3986	19.57894086	4.11×10^{-7}
Stress kinase	Contig20688	484	Receptor protein kinase-like protein	25.87913	29.76964	2.712162	2.535975	24.97827878	5.70×10^{-7}
Stress kinase	Contig23425	314	Leucine-rich repeat protein kinase-like protein	13.01678	8.756681	1.229567	1.814872	5.16838313	3.60×10^{-2}
Stress kinase	Contig41353	346	Receptor protein kinase CLAVATA1, putative	1.778288	3.95121	17.40726	27.53484	5.638999038	4.43×10^{-3}
Stress kinase	Contig43089	3886	Probable LRR receptor-like serine/ threonine-protein kinase	0.169644	0.332042	8.459873	9.618531	0.821163419	2.16×10^{-2}
Stress kinase	Contig7698	471	Receptor protein kinase-like protein	41.19647	35.2224	19.71406	9.213964	16.84079896	3.16×10^{-2}
Stress kinase	Contig9735	2271	Mitogen activated protein kinase 6	35.06641	28.60465	58.15913	62.1221	19.75205629	2.64×10^{-3}
Phytohormone signaling	Contig10455	1130	Auxin induced-like protein	12.56245	9.556372	0.444168	1.189653	5.002390513	5.70×10^{-3}
Phytohormone signaling	Contig17087	1655	Auxin-induced protein 5NG4	5.589903	10.61804	0.594873	3.531612	11.45855049	3.03 × 10−3
Phytohormone signaling	Contig5460	1539	Auxin induced-like protein	24.30195	14.86183	74.75834	113.6777	10.63976556	2.02×10^{-10}
Phytohormone signaling	Contig7592	2980	PIN-like auxin efflux carrier	12.70543	21.11348	6.147548	5.486872	16.88834762	4.72×10^{-3}
Phytohormone signaling	Contig2796	678	GASA5-like protein	38.11517	33.12332	0.113889	0.474137	13.42039175	6.94×10^{-10}
Defence/stress reponse	First_Contig4	772	Disease resistance associated protein	203.1224	277.5106	10.17722	45.21784	260.9048067	5.56×10^{-70}
Defence/stress reponse	Contig68841	366	Senescence-associated protein	148.2983	77.47592	27.58498	381.4702	925.7755612	4.51×10^{-7}
Defence/stress reponse	Contig30151	1234	Late embryogenesis abundant protein	1.656105	0.821571	58.2255	139.7144	2.201737049	1.45×10^{-16}
Defence/stress reponse	Contig3940	433	Putative CC-NBS-LRR protein	87.08625	160.9168	2.318288	14.37582	114.2078959	3.16×10^{-45}
Defence/stress reponse	Contig11348	742	Glutathione peroxidase	56.83178	36.20779	350.9099	117.6447	13.7864769	2.11×10^{-63}
Defence/stress reponse	Contig25275	409	Peroxidas e-like protein	22.45811	24.1117	48.42572	65.91497	9.451633971	1.00×10^{-2}
Defence/stress reponse	First_Contig131	886	Peroxidase	29.66318	46.0133	2745.947	1828.864	49.90825239	0.00
Defence/stress reponse	Contig7174	700	Peroxidase	50.60427	50.84452	626.0631	226.4658	20.13612022	8.49×10^{-124}
Defence/stress reponse	Contig8482	842	Glutathione peroxidase	112.4304	52.632	358.8932	226.2611	32.76583034	3.10×10^{-55}

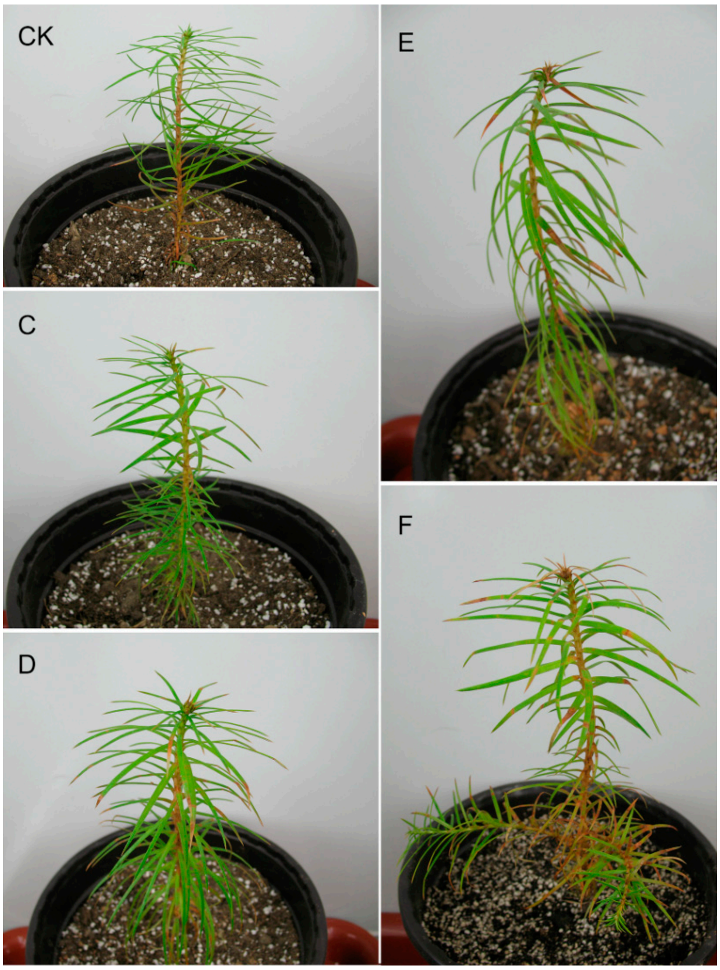

Figure S1. Plantlets used for the transcriptome analysis. CK control, C—5 days drought-treated, D—10 days drought-treated, E—15 days drought-treated, F—24 h post-rewatering.

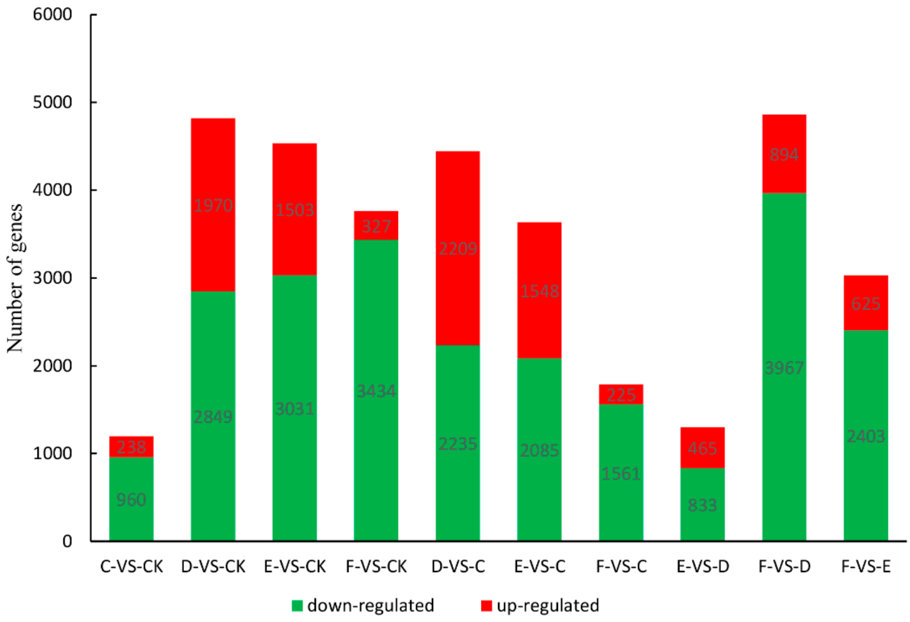

Figure S2. The numbers of up- and down-regulated genes between five libraries (CK, C, D, E, and F).

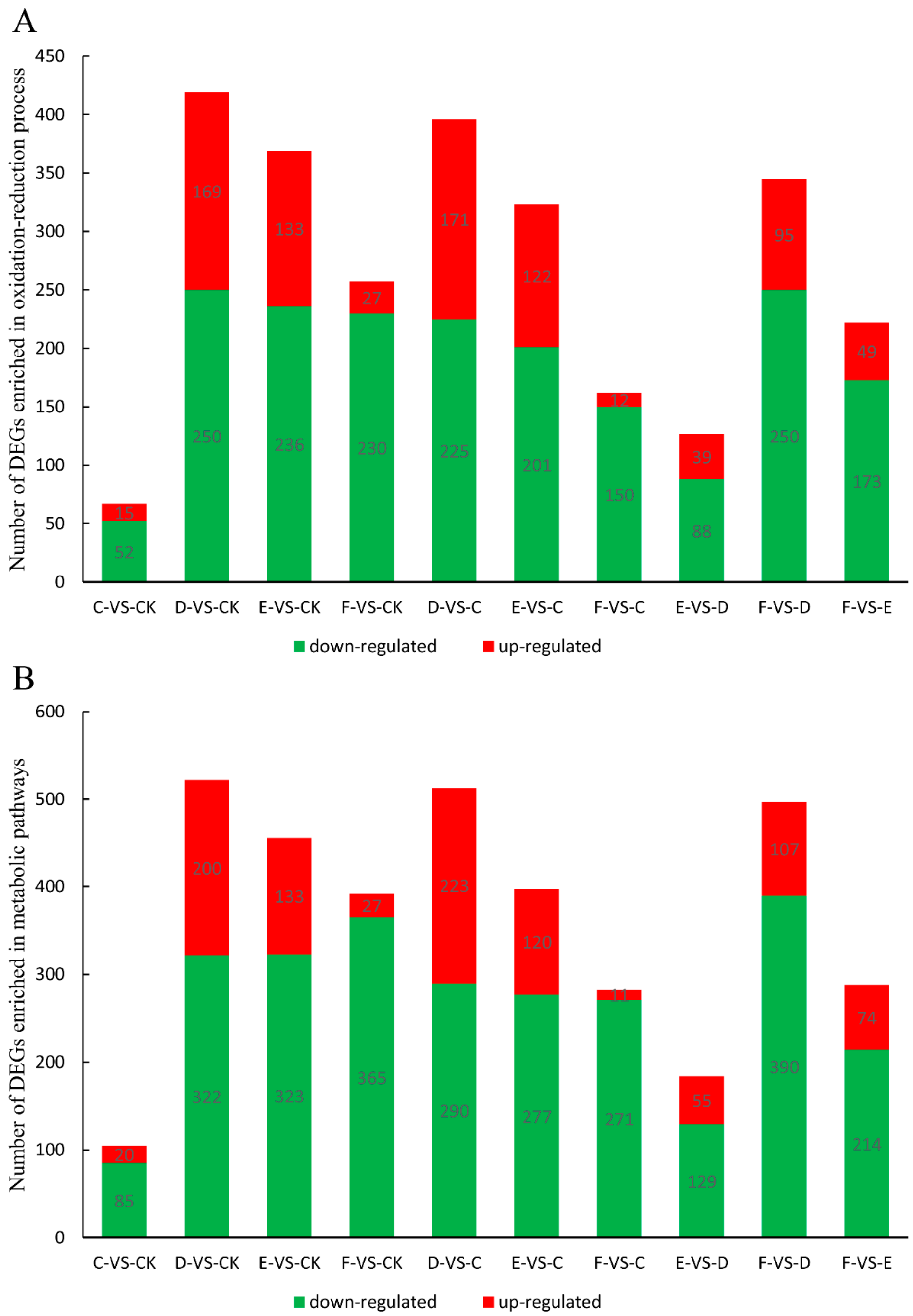

Figure S3. The changes of number of DEGs enriched in GO oxidation-reduction process and KEGG metabolic pathways. CK represents the control; C, D, and E represent the drought treatments; F represents re-watering. The numbers of up- and down-regulated genes among five libraries (CK, C, D, E, and F) are summarised. (**A**) Changes in DEGs enriched in GO oxidation-reduction process among the various drought-stress treatments and re-watering; (**B**) Changes in DEGs enriched in KEGG metabolic pathways among the various drought-stress treatments and re-watering.
